# Validity and reliability of Turkish version of "Hospital Survey on Patient Safety Culture" and perception of patient safety in public hospitals in Turkey

**DOI:** 10.1186/1472-6963-10-28

**Published:** 2010-01-28

**Authors:** Said Bodur, Emel Filiz

**Affiliations:** 1 Department of Public Health and Medical Statistics, Meram Faculty of Medicine, Selcuk University, Konya, Turkey; 2 Public Health Nurse, College of Health, Selcuk University, Konya, Turkey

## Abstract

**Background:**

The Hospital Survey on Patient Safety Culture (HSOPS) is used to assess safety culture in many countries. Accordingly, the questionnaire has been translated into Turkish for the study of patient safety culture in Turkish hospitals. The aim of this study is threefold: to determine the validity and reliability of the translated form of HSOPS, to evaluate physicians' and nurses' perceptions of patient safety in Turkish public hospitals, and to compare finding with U.S. hospital settings.

**Methods:**

Physicians and nurses working in all public hospitals in Konya, a large city in Turkey, were asked to complete a self-administrated patient safety culture survey (n = 309). Data collection was carried out using the Turkish version of HSOPS, developed by Agency for Healthcare Research and Quality (AHRQ). Data were summarized as percentages, means, and SD values. Factor analysis, correlation coefficient, Cronbach's alpha, ANOVA, and t tests were employed in statistical analyses. Items on patient safety were categorized into 10 factors. Factor loadings and internal consistencies of dimension items were high.

**Results:**

Most of the scores related to dimensions, and the overall patient safety score (44%) were lower than the benchmark score. "Teamwork within hospital units" received the highest score (70%), and the lowest score belonged to the "frequency of events reported" (15%). The study revealed that more than three quarters of the physicians and nurses were not reporting errors.

**Conclusion:**

The Turkish version of HSOPS was found to be valid and reliable in determining patient safety culture. This tool will be helpful in tracking improvements and in heightening awareness on patient safety culture in Turkey.

## Background

Patient safety is an important healthcare issue in all countries, rich or poor. There are no data, staff notes, or research related to the safety of patients in Turkey except for a committee on infections in public hospitals. After this study has been completed, the Ministry of Health declared a series of suggestions intended to improve patient and staff safety. This topic is still in its infancy in Turkey; the history of national congresses and symposiums on patient safety dates back only two years prior to this study. State institutions provide Ninety-three percent of all healthcare services in Turkey. Ongoing challenges at state hospitals include lack of primary staff (i.e., physicians and nurses), heavy patient load, and inadequate physical infrastructure. Furthermore, oversight for safety and quality is a concern. The same authority responsible for oversight also provides inspection, so there is a conflict of interest. Further, improvements in patient safety are slow because of public demand for patient rights are rather low.

Medical errors have been identified as one of the five most common causes of death and are at unacceptably high levels [1]. The World Health Organization (WHO) estimates that tens of millions of patients worldwide endure disabling injuries or death each year that can be attributed directly to unsafe medical practices and care [2]. The incidence of medical errors during healthcare procedures is 7.5%, and a majority of the adverse events are identified as preventable [3,4].

One aspect of patient safety that has been increasingly of interest is the "culture" of safety. A *culture *is described as the cumulative result of an entire set of common behaviors, experiences, beliefs, and values that reflects how things are done in a certain environment. To establish a safety culture in a healthcare organization, the first step is evaluating the current culture [5]. Assessment of safety culture in a given institution helps inform the perceptions and behaviors of administrators regarding safety as well as identify the most problematic areas for improvement [6].

High-risk industries--such as commercial aviation and the oil and gas industry--have achieved exemplary safety performance. Safety culture provides an important explanation of such organizations' performance [7]. On the basis of earlier industrial models, future hospital and healthcare studies should examine the effect of safety culture [8-10]. High-reliability organizations are those that operate in a hazardous environment but, from a statistical standpoint, have low rates of adverse events. High-reliability organizations are those where lines of communication remain open to fully share critical information, unfettered by barriers of hierarchy, seniority, title, pay grade, gender, or ethnic background. The healthcare industry is in an ideal position to learn from the experience of aviation and other high-reliability organizations [11].

Safety climate surveys are now being used to measure the safety culture of healthcare organizations, and a comprehensive report of scale development was provided [12]. A survey developed by Agency for Healthcare Research and Quality (AHRQ) is used increasingly in the United States and England [13,14]. It is considered valid, reliable, and the most efficient tools used for patient safety culture [12,15].

This survey includes questions that measure patient safety culture at work area/unit and hospital levels while including outcome variables [16]. Flin [17] state that safety climate questionnaires must achieve as high a standard of measurement as possible so that healthcare managers can rely on resulting data as true indicators of the safety culture. The current study was performed to determine the validity and reliability of the Turkish version of "Hospital Survey on Patient Safety Culture," evaluate the perception of the patient safety among healthcare staff in public hospitals, and compare Turkish data with U.S. data. Patient safety culture is a new field in Turkey, and no study has been published in Turkey on this topic.

## Methods

### Study Group

The study was carried out in 2008 in all the public hospitals--1 general hospital, 1 teaching hospital, and 1 university hospital--located in the metropolitan center of Konya Province, Turkey. Konya is one of the most important cities in central Anatolia. It is world renowned for its history and culture and is famous as an international tourist city.

More than 90% of healthcare services in Turkey are provided by public enterprises. Out of all public hospitals, 775 are general, 59 are teaching, and 57 are university hospitals. Certain general, teaching, and university hospitals have the greatest bed capacity; therefore, this study was carried out in the hospitals that represent these three types of large-capacity hospitals. About 96% of the people in Turkey have social security insurance that provides free health care, and patients are free to choose their hospitals.

Efforts were made to include physicians and nurses--both permanent and temporary--who have direct interaction with patients. The temporary group includes nurses, emergency technicians, and midwives whom they work on nursing services. These workers operate under the direction of supervisors and are under contract for provisional time periods. The size of the study group was determined by a formula that enabled a comparison of the predicted mean score of 63 ± 10 [18] for positive perception of the overall patient safety culture in the hospital within an SD of 5 points, at a 95% confidence level and with a 0.80 power. Thus, the goal was to reach at least 63 people in each group. However, considering the careful attention paid to representing as many units as possible and the likelihood of a low rate of response, the surveys were delivered individually many of the available staff. The response rate varied from 51% to 91%, depending on the profession. The response rate was 51% for doctors, 64% for temporary nurses, and 91% for registered nurses. By hospital type, response rate was 56% for university hospitals, 72% for general public hospitals, and 86% for teaching hospitals. A total of 309 questionnaires were completed and returned as follows: 102 physicians, 135 nurses, and 72 temporary nurses from three public hospitals. The demographics and professional characteristics of the participants are shown in Table 1.

**Table 1:** The demographic and professional characteristics of the participants

	Doctors (n = 102)		R. Nurses (n = 135)		Temp. Nurses (n = 72)	
**Demographic features**	**n**	**%**	**n**	**%**	**n**	**%**

**Gender**						
Male	71	69,6	2	1,5	28	38,9
Female	31	30,4	133	98,5	44	61,1

**Marital status**						
Unmarried	27	25,7	38	28,1	36	50,0
Married	73	72,3	94	69,6	34	47,2
Widowed/divorced	2	2,0	3	2,2	2	2,8

**Type of hospital**						
General public	25	24,5	55	40,7	13	18,1
Teaching	17	16,7	38	28,1	29	40,3
University	60	58,8	42	31,1	30	41,7

**Working Unit**						
Medicine	48	47,1	66	48,9	30	41,7
Surgery	25	24,5	38	28,1	21	29,2
Emergency/ICU/OR*	29	28,4	31	23,0	21	29,2

**Experience in profession**						
Less than 5 year	29	28,4	32	24,4	39	54,9
5 to 9 years	27	26,5	33	25,2	10	14,1
10 to 14 years	15	14,7	34	26,0	12	116,9
15 years or more	31	30,4	32	24,4	10	14,1

**Experience in current hospital work area/unit**						
Less than 1 year	19	18,6	23	17,2	28	38,9
1 to 4 years	42	41,2	45	33,6	29	40,3
5 to 9 years	22	21,6	21	15,7	6	8,3
10 years or more	19	18,6	45	33,6	9	12,5

*ICU: Intensive Care Unit, OR: Operating Room

### Measurement

#### Hospital Survey on Patient Safety Culture (AHRQ)

A Turkish version of by AHRQ's "Hospital Survey on Patient Safety Culture" was developed [16]. The survey was composed of items measuring the dimensions of patient safety culture at work area/unit and hospital levels and included questions measuring outcome variables. Moreover, there were 7 items that asked for personal information. Eighteen of the 42 items belonging to 12 dimensions were worded negatively. The five-level Likert scale was employed for the responses. The scales used for the questions were (1) strongly disagree, disagree, neither, agree, or strongly agree and (2) never, rarely, sometimes, most of the time, or always. Structural validity of each safety culture dimension was partially associated with all others and was reflected by composite scores. This relationship was shown with a correlation between 0.23 and 0.60. Highest correlations were calculated for result variables of "overall perceptions of patient safety" and "patient safety grade" (r = 0.66, p < 0.001). The lowest relationship was revealed by "frequency of events reported" and "non-punitive response to error" (r = 0.23, p < 0,001). The differing nature of safety culture composite scores between hospitals was shown with ANOVA [16].

#### Preparing the Turkish version of the survey

The investigators performed initial translation of the survey into Turkish. An expert in the English language whose native language is Turkish reviewed this translation. Then an independent translator who had not seen the original questionnaire [19] translated it back into English. Sorra, who is one of the authors of the original survey, checked the last translated questionnaire. The Turkish questionnaire was revised according to suggestions on 3 of the 42 items that might have led to differing interpretations. The items of the translated survey were categorized under 10 factors. This might be caused by ambiguity of the translation or cultural differences. Three items were relocated between the dimensions. It was assumed that this state would not affect the general score of the culture of patient safety.

#### Pilot study

The comprehensibility of the survey was tested on 5 physicians and 8 nurses who had not been included in the study group and their opinions were used to prepare the final version of the survey.

#### Data collection

An assistant researcher made an in-person presentation about the surveys to the study group. After participants gave verbal consent, each participant was asked to fill out the survey without discussion.

#### Ethical considerations

The researchers obtained the approval of the ethics committee and obtained institutional permits and consent of the participants.

### Statistical Analyses

The researchers checked to make sure the data set was complete and to determine if the inter-item correlations were sufficient (Bartlett's test χ2 = 4238; df = 861; p < 0.001). There was no high correlation between items. In addition, the Kaiser-Meyer-Olkin measure of sampling adequacy was determined. The KMO-score was 0.9, far above Kaiser's criterion of 0.5. The pre-analyses demonstrated that the data could be used for factor analysis. A confirmative factor analysis was performed (with Varimax rotation) to investigate whether the factor structure of the AHRQ survey could be used with Turkish data. In addition, it was studied using explorative factor analysis to check whether the items formed different factors in the Turkish situation. The construct validity was studied by calculating scale scores for every factor (after any necessary reverse coding) and the correlation coefficients between the scores. The internal consistency of the factors was calculated with Cronbach's alpha.

For each positively worded item, the percentage of positive responses was calculated--that is, the percentage of respondents answering the question by checking "strongly agree" and "agree" or "always" and "most of the time" [16]. The data obtained was summarized as percentage, mean, and SD values. Categorical values were examined using the chi-square test. ANOVA and t tests were applied to compare the mean values, and P < 0.05 was recognized as statistically significant.

## Results

### Factor analysis

The AHRQ survey is comprised of 12 dimensions. The Turkish version included 42 items that resulted in 10 factors. Therefore, the number of items under some factors changed to original distribution. However, since the factor loading of each item was above 0.40, the structure of the Turkish survey was considered appropriate. Additionally, the internal consistency was calculated for every factor and compared with the internal consistency found in the AHRQ study. The internal consistency of the Turkish items was lower for each factor than the original items in the AHRQ study except for frequency of events reported (α = 0.86) and teamwork within units (α = 0.83). The internal consistency of two factors was poor: staffing (α = 0.19) and non-punitive response to error (α = 0.31).

The 10 factors were identified using explorative factor analysis (Table 2). While "feedback and communication about error" and "management expectations and actions promoting patient safety" dimensions were grouped under one factor, "teamwork across units" and "management support for patient safety" dimensions were grouped under another factor. Factor loadings were between 0.36 and 0.87. The factors jointly explain 62.1% of the variance in the responses. The internal consistency was calculated for every factor according to the original dimensions and items. Except in two dimensions, the Cronbach's alpha coefficient was between 0.57 and 0.86. Internal consistency reliability for all items was high (α = 0.88). Moreover, the Spearman-Brown coefficient was 0.84, and the Guttman split-half coefficient was 0.80.

**Table 2:** Factor loadings of the items regarding patient safety culture

Items	F1	F2	F3	F4	F5	F6	F7	F8	F9	F10
We are informed about errors that happen in this unit	**0,68**									
Staff feel free to question the decisions or actions of those with more authority	**0,66**									
Staff will freely speak up if they see something that may negatively affect patient care	**0,60**									
Staff are afraid to ask questions when something does not seem right	**0,60**									
We are given feedback about changes put into place based on event reports	**0,56**									
In this unit, we discuss ways to prevent errors from happening again	**0,54**									
Shift changes are problematic for patients in this hospital		**0,68**								
Important patient care information is often lost during shift changes		**0,68**								
Things fall between the cracks when transferring patients from one unit to another		**0,61**								
It is often unpleasant to work with staff from other hospital units		**0,58**								
Problems often occur in the exchange of information across hospital units		**0,46**								
Staff in this unit work longer hours than is best for patient care			**-0,73**							
After we make changes to improve patient safety, we evaluate their effectiveness			**0,51**							
We are actively doing things to improve patient safety			**0,49**							
People support one another in this unit				**0,77**						
In this unit, people treat each other with respect				**0,77**						
When one area in this unit gets really busy, others help out				**0,72**						
When a lot of work needs to be done quickly, we work together as a team to get the work done				**0,62**						
Hospital management provides a work climate that promotes patient safety					**0,69**					
Hospital units do not coordinate well with each other					**0,65**					
There is good cooperation among hospital units that need to work together					**0,63**					
The actions of hospital management show that patient safety is a top priority					**0,62**					
Hospital units work well together to provide the best care for patients					**0,57**					
Hospital management seems interested in patient safety only after an adverse event happens					**0,55**					
Mistakes have led to positive changes here						**0,66**				
When an event is reported, it feels like the person is being written up, not the problem						**0,84**				
Staff feel like their mistakes are held against them						**-,54**				
Staff worry that mistakes they make are kept in their personnel file						**-,38**				
We use more agency/temporary staff than is best for patient care							**-,80**			
We have enough staff to handle the workload							**0,61**			
We work in crisis mode, trying to do too much, too quickly							**0,55**			
Our procedures and systems are good at preventing errors from happening								**0,57**		
It is just by chance that more serious mistakes don't happen around here								**0,54**		
Patient safety is never sacrificed to get more work done								**0,51**		
We have patient safety problems in this unit								**0,40**		
When a mistake is made, but has no potential to harm the patient, how often is this reported?									**0,89**	
When a mistake is made, but is caught and corrected before affecting the patient, how often is this reported?									**0,87**	
When a mistake is made that could harm the patient, but does not, how often is this reported?									**0,78**	
My supervisor/manager seriously considers staff suggestions for improving patient safety										**0,76**
My supervisor/manager says a good word when he/she sees a job done according to established patient safety procedures										**0,76**
My supervisor/manager overlooks patient safety problems that happen over and over										**0,51**
Whenever pressure builds up, my supervisor/manager wants us to work faster, even if it means taking shortcuts										**0,36**

### Construct Validity

For each of the 10 factors, scale scores were calculated by obtaining the mean of the item scores within one factor for every respondent. Next, correlations between the scale scores were calculated. The highest correlation was between "teamwork across units and management support" and "handoffs and transitions" (r = 0.53), but no correlation was exceptionally high (Table 3).

**Table 3:** Inter-correlations of the 10 factors

Factor	1	2	3	4	5	6	7	8	9
**1**. Teamwork within units									
**2**. Management Expectations	0,306**								
**3**. Teamwork across units and Management support	0,335**	0,462**							
**4**. Organizational learning	0,247**	0,289**	0,329**						
**5**. Overall perceptions of safety	0,387**	0,344**	0,408**	0,358**					
**6**. Feedback and communication openness about error	0,382**	0,507**	0,517**	0,355**	0,441**				
**7**. Frequency of events reported	0,132*	0,207**	0,315**	0,289**	0,217**	0,358**			
**8**. Staffing	0,043	0,098	0,114	0,058	0,166**	0,056	-0,032		
**9**. Handoffs and transitions	0,323**	0,314**	0,532**	0,185**	0,353**	0,427**	0,280**	0,04	
**10**. Non-punitive response to error	0,058	0,256**	0,283**	0,158**	0,193**	0,288**	0,122*	0,159**	0,225**

*p < 0.05 **p < 0.01.

Additionally, correlations of the scales with the mono-item outcome variable patient safety grade were calculated. Patient safety grade is a single-item measure--a hospital is graded on overall patient safety. All correlations with the patient safety grade were significant except dimensions of "staffing" and "non-punitive response to error." The highest correlation of this outcome measure was for overall perceptions of safety (r = 0.46). Safety culture may vary among hospitals, and in the present study, differences did occur among the hospitals regarding some dimensions.

### Survey Findings

The mean age of healthcare personnel who participated in the patient safety culture hospital survey was 31 ± 7. While 144 (46.6%) of them were working in the Department of Internal Medicine, 84 (27.2%) were working in the Department of Surgery, and 81 (26.2%) were working in the Intensive Care/Emergency/Operation Room. Healthcare personnel working hours of 65% were between 40 and 49 hours a week. Of the participants, 33% were male, 67.5% female, 33% single, and 67.5% were married. Thirty percent worked at a general public hospital, 27% at a teaching hospital, and 43% at a university hospital. Length of time worked varied, with 33.5% having worked for 5 years or less, and 34% having professional experience of 15 years or longer.

The overall patient safety culture score was 44%. The overall score means were: 38 ± 20% for doctors, 43 ± 19% for registered nurses, and 47 ± 20% for temporary nurses (p = 0.019). Scores were lower in doctors than for temporary nurses (p < 0.05). Mean scores were 40 ± 18% in a university hospital, 39 ± 20% in a general hospital, and 48 ± 22% in a teaching hospital (p < 0.05). There was a difference in general score by gender (p > 0.05), but the safety culture scores were higher in married persons than in others (p < 0.05).

The highest percentage of positive responses was obtained from the "teamwork within units" dimension, whereas items in the "frequency of events reported" dimension received the lowest percent of positive responses. There were no differences in the three types of hospitals regarding most dimensions of the patient safety culture; however, the percentage of positive responses was low in two dimensions and four items in the university hospital. Regarding the profession, one dimension and five items received a lower percentage of positive responses from physicians. In terms of work-area, scores of the Emergency/Intensive Care/Operating Room personnel were lower in two dimensions and six items compared with those who worked in the other departments. People who worked more than 50 hours per week (n = 77), exhibited lower scores in 5 of the dimensions. A comparison of the mean value of the three hospitals for patient safety culture perception with a benchmark score revealed lower mean positive response values in 6 of 10 dimensions and higher in one. *The biggest differences were found in "frequency of events reported" and "management support for patient safety" dimensions *(Table 4).

**Table 4:** Descriptive statistics of survey on patient safety culture and benchmark scores

Ten factors subscales and survey items	Score (SD)	Benchmark score
**Teamwork within units ***(Cronbach's α = 0.83)*	**70 (6)**#	**75**
a. People support one another between units	65(6)	81*
b. When a lot of work needs to be done quickly, we work together as a team to get the work done	75(5)	81*
c. In all units, people treat each other with respect	72(3)	73
d. When one area in this unit gets really busy, others help out	70(9)	63
**Management Expectations and Actions Promoting Patient Safety ***(Cronbach's α = 0.72)*	**44 (6)§**	70*
a. Management says a good word when he/she sees a job done according to established resident safety procedures	37(5)**§**	68*
b. Management seriously considers staff suggestions for improving resident safety	41(7)	72*
c. Whenever pressure builds up, my manager wants us to work faster, even if it means taking shortcuts (R)	37(5)	67*
d. My manager overlooks resident safety problems that happen over and over (R)	60(8)	71*
**Teamwork across units and Management support for resident safety ***(Cronbach's α = 0.80)*	**40 (8)**	62*
a. Management provides a work climate that promotes resident safety	40(12)†‡	72*
b. The actions of management show that resident safety is a top priority	37(6)‡	65*
c. Management seems interested in resident safety only after an adverse event happens	32(4)	50*
d. There is good cooperation among units that need to work together	46(12)	34
e. Units work well together to provide the best care for residents	40(6)	47
f. Units do not coordinate well with each other (R)	57(2)	51*
**Organizational learning ***(Cronbach's α = 0.68)*	**41 (7)**#	66*
a. We are actively doing things to improve resident safety	51(5)‡	77*
b. Staff in this facility work longer hours than is best for resident care	21(4)	48*
c. After we make changes to improve resident safety, we evaluate their effectiveness	52(7)**‡ §**	62*
**Overall perceptions of safety ***(Cronbach's α = 0,57)*	**62 (7)**#	**56**
a. Resident safety is never sacrificed to get more work done	67(7)†	53*
b. Our procedures and systems are good at preventing errors from happening	67(11)	55
c. It is just by chance that more serious mistakes don't happen around here	57(5)**§**	52
d. We have patient safety problems in this facility (R)	57(5)	64*
**Feedback and communication openness about error ***(Cronbach's α = 0.81)*	**38 (6)§**#	59*
a. We are given feedback about changes put into place based on event reports	30(4)**§**	53*
b. We are informed about errors that happen in the units	47(9)	59*
c. In this facility, we discuss ways to prevent errors from happening again	42(6)**§**	65*
a. Staff will freely speak up if they see something that may negatively affect resident care	43(1)† **‡**	72*
b. Staff feel free to question the decisions or actions of those with more authority	19(3)	46*
c. Staff are afraid to ask questions when something does not seem right (R)	47(4)	60*
**Frequency of events reported ***(Cronbach's α = 0.86)*	**15 (4)**	54*
a. When a mistake is made, but is caught and corrected before affecting the resident, how often is this reported?	13(2)	47*
b. When a mistake is made, but has no potential to harm the resident, how often is this reported?	14(3)	50*
c. When a mistake is made that could harm the resident, but does not, how often is this reported?	18(4)**‡**	67*
**Staffing ***(Cronbach's α = 0.63)*	**44 (9)**†	**48**
a. We have enough staff to handle the workload	36(19)† **§**	45
c. We use more agency/temporary staff than is best for resident care	76(9)	61*
d. We work in "crisis mode" trying to do too much, too quickly (R)	21(4)	40*
**Handoffs and transitions ***(Cronbach's α = 0.72)*	**54 (4)† ‡**	35*
a. Things "fall between the cracks" when transferring residents from one unit to another (R)	44(6)	29*
b. Important resident care information is often lost during shift changes (R)	59(3)	44*
c. Problems often occur in the exchange of information across units (R)	42(5)	32*
d. It is often unpleasant to work with staff from other units (R)	46(7)	56*
e. Shift changes are problematic for residents in this facility (R)	67(1)	36*
**Non-punitive response to error ***(Cronbach's α = 0.71)*	**24 (4)**	37*
a. Staff feel like their mistakes are held against them (R)	14(1)	44*
b. When an event is reported, it feels like the person is being written up, not the problem (R)	19(1)	39*
c. Mistakes have led to positive changes here	42(11)	58*
d. Staff worry that mistakes they make are kept in their personnel file (R)	23(4)	28*

**Overall ***(Cronbach's α = 0.86)*	**44 (5)†**	67*

R = item was reverse coded

*Significantly different t test at p,0.05 (using same SD for both data)

**† **Lover in university hospital to general and teaching hospital (p < 0.001)

**‡ **Lower in physicians to nurses (p < 0.05)

**§**Lower in staff working in Emergency/ICU/OR (p < 0.05)

# Lower in staff working 50 hour or more per week (p < 0.05)

While 86% of healthcare personnel gave positive--46% acceptable, 40% good-excellent--responses for "patient safety grade," there was no difference among the hospital types in terms of safety grade (p > 0.05). An important study finding was that a majority of the healthcare personnel (84%) *never *report medical errors relating to patient safety. The frequency of events reported was similar among the types of hospitals (p > 0.05). No difference could be found among the professions or work-areas regarding frequency of events reported (p > 0.05).

## Discussion

Self-report instruments are commonly used, although the weaknesses of self-reporting are widely recognized. Self-report inventories are often a good solution when researchers need to administer a large number of tests in a relatively short period of time. Scoring of the tests is standardized and based on previously established norms. However, self-report inventories have their weaknesses: Some tests are long and tedious. In some cases, a respondent may simply lose interest and not answer questions accurately. Additionally, people are sometimes not the best judges of their own behavior. Some individuals may try to hide their true feelings, thoughts, and attitudes.

The safety culture environment is considered the most important barrier to improving patient care safety [20]. The starting point for developing a safety culture should be the evaluation of the current culture by using an appropriate instrument [21]. This is a starting point for several areas: (1) diagnosis of safety culture and raising awareness, (2) evaluation of patient safety interventions and tracking change over time, (3) internal and external benchmarking, and (4) fulfillment of regulatory or other requirements [6].

In the 10-factor model, the reliability (internal consistency) of the factors and construct validity are acceptable. This indicates that the dimensions measure different constructs. However, high factor loading in all the items and a moderately high correlation among them indicate that each item could be used independently to determine patient safety; the number of items measuring the same factors could be limited to shorten the instrument. Internal consistency (α) coefficients, which are based on the original factor structure of the survey, are shown in Table 4. As expected, all factors were correlated with the outcome variable (i.e., patient safety score). The correlation of the patient safety score with "overall perception of safety" is an indication that the latter scale is accurate.

Overall, we generally found low patient safety culture scores in Turkey. The patient safety culture perception levels of physicians and nurses were similar. The number of participation of nurses in the study was higher than that reported in several other studies [13,22,23]. Improvements may be realized through the following: (1) reporting of adverse events, (2) non-punitive policies with respect to error reporting, (3) open communication, (4)management support for safety culture, and (5) staffing improvements (Table 4).

Personnel appeared unwilling to work with those in other units but reported good teamwork within their own units (Table 4). Other studies found similar results [22,24]. Teamwork is an important part for the development of patient safety, and personnel should be encouraged and supported in their efforts to establish good relationships with people working in other units [1,25].

Frequency of events reported, feedback about error, and organizational learning levels were all quite low (Table 4). In various types of hospitals and in rest homes, personnel surveyed by other researchers expressed concerns about application of punitive approaches [13,24,26]. In a study by Kim and colleagues, nurses were found to be inattentive to the "possible occurrence" of medical error, and a lack of open communication was reported [25]. If there is no system in place to report events and personnel have an intensive workload, reporting of events will be difficult [27]. Some studies reveal higher percentages of positive opinions about organizational learning, event reporting and open communication [23,28].

Management can show its support for patient safety by maintaining open communication, educating personnel, delegating the workers to identify and correct risks, stating that patient safety is a shared responsibility, and providing adequate resources [7]. This study shows that physicians and nurses consider management support to be inadequate for patient safety (Table 4). While there are studies that report similar results [23,25,26,29], there are other studies in which personnel report that management support was adequate [28]. Scarcity in number of personnel and inexperience will weaken the ability of a healthcare facility to provide patient safety.

If healthcare personnel's perception of patient safety culture is generally lower than the benchmark score [14] in hospitals with 400 or more beds, they will also have higher scores in dimensions such as "overall perception of patient safety" and "handoffs and transitions" (Table 4). While this can be due to cultural differences, it can also be due to the reluctance of healthcare personnel to express negative opinions about their workplaces. In other studies that report a low patient safety culture level, "overall perception of patient safety" and "handoffs and transitions" dimensions were also problematic [13,24,26]. Alterations in communication mechanisms during handoffs and a lack of standards in doing so cause patient safety problems [30].

## Conclusions

The factor structures of the Turkish and American "Hospital Survey on Patient Safety Culture" are almost identical. The main part of the factor structure is the same, and all items were kept. There are only small shifts among items across factors. Undeniably, Turkish factors show a lower internal consistency than those in the American study, excluding only two factors. This study demonstrates that this translated "Hospital Survey on Patient Safety Culture" is an appropriate instrument to assess patient safety culture in Turkish hospitals.

Determining the patient safety culture level should be a continuous process. Hospitals in Turkey need to continue to make improvements to their patient safety culture. The first step should be obtaining the support of the administration and assuming a non-punitive approach to those who make and report medical errors. If the problem of personnel not reporting events is to be resolved, any barriers to reporting should be identified and addressed.

## Competing interests

The authors declare that they have no competing interests.

## Authors' contributions

SB performed the statistical analyses and interpretation of the data, and wrote the manuscript. EF coordinated the translation procedure of the questionnaire, collected the data, and contributed to interpretation of the data. SB and EF read and approved the final manuscript.

## Pre-publication history

The pre-publication history for this paper can be accessed here:

http://www.biomedcentral.com/1472-6963/10/28/prepub
